# The burden of orthopaedic disease presenting to a tertiary referral center in Moshi, Tanzania: a cross-sectional study

**DOI:** 10.11604/pamj.2022.42.96.30004

**Published:** 2022-06-06

**Authors:** William Mack Hardaker, Mubashir Jusabani, Honest Massawe, Anthony Pallangyo, Rogers Temu, Gileard Masenga, Nathaniel Fessehaie, Anchi Numfor, Matthew Winterton, Ajay Premkumar, Neil Perry Sheth

**Affiliations:** 1 Duke University Medical Center, Department of Orthopaedic Surgery, Durham, North Carolina, United States of America,; 2 Kilimanjaro Christian Medical University College, Department of Orthopaedics and Traumatology, Moshi, Kilimanjaro, Tanzania,; 3 University of Pennsylvania, Perelman School of Medicine, Philadelphia, United States of America,; 4 University of Pennsylvania, Department of Orthopaedic Surgery, Philadelphia, United States of America,; 5 Hospital for Special Surgery, Department of Orthopaedic Surgery, New York, United States of America

**Keywords:** Road traffic trauma, epidemiology, trauma, injury, orthopaedics

## Abstract

**Introduction:**

as road traffic crashes (RTCs) continue to rise in the developing world, the current growth rate and true burden of orthopaedic injuries are unknown. In 2015, we characterized the orthopaedic burden at Kilimanjaro Christian Medical Center (KCMC) in Tanzania. In this study, we re-evaluated the burden and growth-rate over three years in the absence of any system level changes. Additionally, we calculated the percentage of orthopaedic patients that received definitive fixation for their orthopaedic injury when surgery was indicated.

**Methods:**

we prospectively collected data for 190 patients admitted to the orthopaedic ward at KCMC during June/July 2018. We also retrospectively reviewed available records for patients presenting to the KCMC Emergency Department, Orthopaedic Outpatient Clinic and Orthopaedic Ward.

**Results:**

prospective data: 231 patients were admitted to the orthopaedic ward. Forty-one (17.7%) isolated spine patients were excluded, leaving 190 patients in the final study cohort. RTC (89, 46.8%) represented the most common mechanism of injury requiring orthopaedic ward admission, followed by falls (60, 31.6%) and infections (14, 7.4%). Femur fractures were the most common injury (62, 31.0%), followed by tibia fractures (27, 13.5%), isolated fibula fractures (23, 11.5%), and foot fractures (23, 11.5%). Almost 96% of admitted patients were indicated for surgical fixation, but only 44.5% received definitive fracture treatment. Retrospective data: KCMC treated an average of 15,117 orthopaedic patients per year, representing a 35.3% growth in the orthopaedic burden compared to 2015.

**Conclusion:**

the burden of orthopaedic surgical disease at KCMC is increasing. Without innovative strategies to address this situation, the discrepancy between the need for orthopaedic care and surgical care capacity at KCMC and in similar settings will continue to grow.

## Introduction

The burden of musculoskeletal disease in low- and middle-income countries (LMIC) continues to increase mainly due to road traffic crashes (RTC). Globally, RTCs constitute over 1.35 million deaths annually - more deaths than HIV/AIDS, tuberculosis and diarrheal diseases combined, and represent the third leading cause of disability for people aged 15-44 [1,2]. Traumatic musculoskeletal injuries often necessitate orthopaedic surgical treatment. However, in LMICs, even at tertiary referral centers, definitive care is not readily accessible for most patients. Countries in sub-Saharan East-Africa are no exception to this scenario. In 2017, Tanzania experienced 17,840 deaths due to RTCs, the 9^th^ highest mortality rate from RTCs worldwide [2]. For its population of almost 50 million people, there are 45 consultant orthopaedic surgeons; only one orthopaedic surgeon for every 1.1 million Tanzanians [3]. Given this massive discrepancy, the current workforce is unable to address the demand for orthopaedic services.

Our group previously characterized the orthopaedic burden at Kilimanjaro Christian Medical Center (KCMC) in Northern Tanzania. At this tertiary referral center, the orthopaedic volume is comparable to that of a level one trauma center in the United States of America (USA), and only 10% of the catchment area population have access to orthopaedic surgical care [4,5]. As RTCs continue to rise in the developing world, the current growth rate and true burden of orthopaedic injuries is still unknown [1]. In this study, we calculated the percentage of orthopaedic patients that received definitive fixation for their orthopaedic injury when surgery was indicated. We also re-evaluated KCMC´s orthopaedic burden and documented the growth-rate since 2015 in the absence of any system level changes. We hypothesized that the number of patients that received definitive treatment of their musculoskeletal injury would be less than previously reported and that the burden of disease at KCMC would grow considerably over a three-year period.

## Methods

**Setting:** KCMC is a 700-bed facility in Northern Tanzania and is one of the country´s four large tertiary referral centers, with a catchment area covering 12.5 million people. KCMC has limited material and personnel resources, including just four full-time orthopaedic surgeons. The orthopaedic ward consists of 66 total beds. There are five operating theaters of which only one is dedicated to orthopaedic surgery.

**Study design:** we prospectively collected data for patients admitted to the orthopaedic ward from June to July 2018 [4]. We also retrospectively reviewed available records for patients presenting to the emergency department (ED), orthopaedic outpatient clinic and orthopaedic ward (Table 1). This study received KCMC Clinical Research Ethical Review Committee approval prior to data collection and analysis (Ethical Clearance Certificate number 2220). Data was de-identified during collection and stored in a password protected database.

**Table 1:** retrospective data sources and study periods

Patient record type	Time period	Dates
Emergency department	Seven consecutive months	October 1, 2017 - April 30, 2018
Orthopaedic outpatient clinic	81 consecutive clinic days	April 10, 2018 - August 1, 2018
Orthopaedic ward admissions	31 consecutive months	January 1, 2016 - July 31, 2018

### Data collection

**Prospective data:** all patients, except those with isolated spine injuries, admitted to the orthopaedic ward during the study period were included for prospective data collection. Data collected included age, sex, region of residence, occupation, mechanism of injury, diagnosis, fracture location, fracture type (open vs closed), pre-operative treatment, surgery provided, time to surgery, and hospital length of stay. Radiographs were reviewed by two authors (MJ and AP), classified according to 2018 *Arbeitsgemeinschaft für Osteosynthesefragen*/ Orthopaedic Trauma Association (AO/OTA) Guidelines and it was determined if surgical fixation was indicated [6]. Post-operative radiographs were evaluated for the presence of definitive fixation. Patients who received open reduction and internal fixation (ORIF) or intramedullary nailing were categorized as having undergone definitive surgical fracture treatment; cases for which external fixation was used as definitive treatment were individually assessed for appropriateness. Implant removal procedures were categorized as definitive treatment.

**Retrospective data:** all available emergency department (ED), outpatient clinic and orthopaedic ward records were reviewed by two authors (WH and MJ). Review of ED records established the total number of orthopaedic consultations. Outpatient analysis determined the total number of evaluated clinic patients and the percentage of patients presenting with health insurance. Retrospective review of orthopaedic ward data determined the number of ward admissions, diagnoses and discharge status (including deaths).

**Statistical analysis:** statistical analysis was performed using the R: a language and environment for statistical computing. Student t-tests were performed for continuous data and χ^2^ or Fisher exact tests were performed for categorical data. Two-tailed p values <0.05 were considered statistically significant. Cohen´s Kappa statistic was calculated to assess inter-observer agreement of prospective definitive treatment designations and inter-observer agreement of retrospective data abstraction.

## Results

**Prospective cohort:** during the study period, 231 patients were admitted to the orthopaedic ward. Forty-one (17.7%) isolated spine patients were excluded, leaving 190 patients in the final study cohort. The majority of admitted patients were male and under the age of 45 years (Table 2). Males tended to be younger (mean age 37.8, range 3 to 98 years, SD 23.7) than females (mean age 43.9, range 2 to 94 years, SD 21.5) (p = 0.108) (data not shown). The most common occupations were farmer, businessman and student, which together accounted for over 50% of patient reported jobs. Of the 190 patients prospectively followed during the six-week study period, 138 (72.6%) were discharged home, 45 (23.7%) were still admitted at the end of the data collection period and seven (3.7%) died as an inpatient.

**Table 2:** demographic data (age, sex, occupation) (n=190)

Variable	n	%
**Age, years**		
0-14	23	12.1
15-44	104	54.7
45-64	37	19.5
More than 64	26	13.7
Age, years, mean (SD)	39.2 (22.1)	
Sex, male	147	77.4
**Occupation**		
Farmer	38	20.0
Business	31	16.3
Student	31	16.3
Field worker	26	13.7
Driver	15	7.9
Tradesman	12	6.3
Unemployed	10	5.3
Other	27	14.2

Road traffic crashes (RTC) (89, 46.8%) represented the most common mechanism of injury requiring orthopaedic ward admission, followed by falls (60, 31.6%) and infections (14, 7.4%) (Table 3). Motorized vehicles versus pedestrians represented 28.1% (25/89) of the RTC group. The majority of RTCs (61, 68.5%) were motorcycle-related (motorcycle driver/passenger or pedestrian struck by motorcycle). The most common reason for admission was fracture (148, 77.9%), followed by infection (16, 8.4%) and removal of implant (ROI) (8, 4.2%).

**Table 3:** injury characteristics (mechanism, reason for admission) (n=190)

Variable	n	%
**Mechanism of injury**		
RTC	89	46.8
Motorcycle	47	52.8
Pedestrian	25	28.1
Car	11	12.4
Truck	3	3.4
Bicycle	2	2.2
Bus	1	1.1
Falls	60	31.6
Infection	14	7.4
Tumor/mass	10	5.3
Assault	7	3.7
Crush injury	6	3.2
Other	4	2.1
**Reason for admission**		
Fracture	148	77.9
Infection	16	8.4
Removal of implant	8	4.2
Tumor/mass	7	3.7
Dislocation	4	2.1
Laceration	3	1.6
Muscular injury	3	1.6
Gout	1	0.5

RTC: road traffic crash

Of the patients diagnosed with a fracture, 34.5% (51) had at least one open fracture while 65.5% (97) had only closed fractures. Femur fractures were the most common injury (62, 31.0%), followed by tibia fractures (27, 13.5%), isolated fibula fractures (23, 11.5%), and foot fractures (23, 11.5%) (Table 4). One-hundred twenty-four (83.8%) fracture patients had a lower extremity fracture. Fifty-six (37.8%) fracture patients sustained multiple fractures; thirty-three (22.3%) patients had fractures involving multiple extremities. X-rays were available for 122/148 (82.4%) fracture patients, who collectively sustained a total of 200 fractures.

**Table 4:** AO/OTA fracture classification - prospective admissions (n=200)

Fracture	AO/OTA classification	n	%
**Femur**		**62**	**31.0%**
Trochanteric region	31A	13	21.0%
Femoral neck	31B	12	19.4%
Femoral head	31C	0	0.0%
Diaphyseal - simple	32A	18	29.0%
Diaphyseal - wedge	32B	10	16.1%
Diaphyseal - multi-fragmentary	32C	3	4.8%
Distal extraarticular	33A	2	3.2%
Partial articular	33B	1	1.6%
Complete articular	33C	2	3.2%
**Tibia**		**27**	**13.5%**
Proximal articular	41	5	18.5%
Diaphyseal	42	20	74.1%
Distal articular	43	2	7.4%
**Fibula**		**23**	**11.5%**
Proximal articular	4F1	4	17.4%
Diaphyseal	4F2	18	78.3%
Distal articular	4F3	1	4.3%
**Foot**		**23**	**11.5%**
Metatarsal	87	15	65.2%
Phalanx	88	6	26.1%
Talus	81	1	4.3%
Foot crush injury	89	1	4.3%
**Radius**		**12**	**6.0%**
Proximal articular	2R1	0	0.0%
Diaphyseal	2R2	6	50.0%
Distal articular	2R3	6	50.0%
**Ankle**		**11**	**5.5%**
Infra-syndesmotic fibula injury	44A	0	0.0%
Trans-syndesmotic fibula fracture	44B	3	27.3%
Supra-syndesmotic fibula fracture	44C	8	72.7%
**Pelvis**		**9**	**4.5%**
Acetabulum	62	4	44.4%
Pelvic ring	61	5	55.6%
**Humerus**		**9**	**4.5%**
Proximal articular	11	2	22.2%
Diaphyseal	12	2	22.2%
Distal articular	13	5	55.6%
**Hand**		**9**	**4.5%**
Metacarpal	77	7	77.8%
Phalanx	78	1	11.1%
Scaphoid	72	1	11.1%
**Ulna**		**7**	**3.5%**
Proximal articular	201	0	0.0%
Diaphyseal	202	4	57.1%
Distal articular	203	3	42.9%
**Other**		**8**	**4.0%**
Patella	34	5	62.5%
Rib	16	2	25.0%
Clavicle	15	1	12.5%

The average time from admission to surgical intervention was 4.2 days (range, 0 to 40 days, SD 6.2). Of the patients who were discharged during the study period, the mean hospital length of stay was 11.6 days and the median length of stay was 6 days (range, 1 to 46 days, SD 7.0). Few non-surgical patients were admitted during the study period (8/190, 4.2%); the majority were pediatric patients with a mid-shaft femur fracture that were treated with plaster of Paris casting. Among patients indicated for surgical intervention (131/182, 72.0%), less than half received definitive fracture treatment (81/182, 44.5%).

Retrospective cohort: from October 1, 2017 through April 30, 2018, 1,271 orthopaedic consultations were evaluated in the ED; this corresponded to an average of 6 consultations per day, 182 consultations per month, and 2,179 per year. Over 81 consecutive clinic days from April 10, 2018 through August 1, 2018, 3,802 patients were evaluated in the outpatient orthopaedic clinic; this corresponded to an average of 47 patients per day, 235 patients per week, 939 patients per month, and 11,266 patients per year. A majority of clinic patients 61.3% (2,330) had some form of health insurance, while all other patients (1,471) paid out of pocket. The out-of-pocket expense was approximately $2.20; depending on the services provided, some were required to pay as much as $22.00. As benchmark reference, the gross domestic product (GDP) per capita in Tanzania per week is $19.14 [7].

During the 31 consecutive months from January 1, 2016 through July 31, 2018, 4,318 patients were admitted to the orthopaedic ward. This corresponded to an average of 139 patient admissions per month and 1,672 admissions per year. The kappa coefficient for retrospective data collection was 0.96, indicating excellent inter-observer reliability between the two reviewers.

Based on the annualized data presented above, KCMC treated an average of 15,117 orthopaedic patients per year. Approximately 74.5% (11,266) were seen as outpatients in clinic, 14.4% (2,179) were treated and discharged from the ED, and 11.1% (1,672) were admitted to the inpatient orthopaedic ward. Relative to 2015, ED consultations decreased by 35.1%, while outpatient evaluations and inpatient admissions increased by 76.6% and 16.9%, respectively. Between 2015 and 2018, the overall number of musculoskeletal patients at KCMC increased by 35.3% (Table 5).

**Table 5:** retrospective data - EMD consultations, orthopaedic clinic patients, and orthopaedic ward admissions in 2015 and 2018

Clinical setting	2015	2018	Percentage change
EMD consultations	3358	2179	-35.1%
Orthopaedic clinic patients	6379	11266	+76.6%
Orthopaedic ward admissions	1430	1672	+16.9%
Total patients	11172	15117	+35.3%

EMD: emergency medical dispatch; 2018 data represents annualized data from the corresponding retrospective data collection periods

## Discussion

Our objective in this study was to evaluate the growth of the burden of orthopaedic disease presenting to KCMC over a three-year period and to provide a measurable assessment of the current system´s ability to meet this burden. We documented a 35% increase in orthopaedic burden and that over 95% of prospective patients admitted to the Orthopaedic Ward at KCMC were indicated for operative intervention; this is in line with our previous findings [4]. In 2015, 57.9% of surgical candidates were taken to the operating room for treatment. However, this group included both patients who received temporizing treatments such as bedside irrigation or traction pin placement and those that received definitive treatment (ORIF, intramedullary nailing or removal of implant). In the current study, we sub-categorized patients into two groups: temporizing treatment and definitive fixation. While over 70% of surgical candidates were taken to the operating room for any surgical treatment, only 44.5% of the total received definitive treatment for their fracture. We believe that by excluding procedures that did not provide definitive treatment, this percentage is a more accurate reflection of the current surgical system´s ability to definitively treat orthopaedic patients.

Several surgical system constraints prevent the delivery of definite fracture treatment including: 1) The patient´s inability to pay for surgery; 2) a lack of a steady supply of expensive orthopaedic implants and; 3) an inadequate number of operating theaters, which compromises surgical capacity. The lack of affordability is a major constraint. For the operative procedure alone, the out-of-pocket cost at KCMC is over $100 (230,000 TZS as per the foreign exchange rate on 5/26/2020), significantly more than the average Tanzanian monthly income. Additional fees for implants, imaging, medications, and hospital stay financially overwhelm patients and their family. Our group´s previous work at KCMC revealed that nearly 75% of orthopaedic patients reported catastrophic healthcare expenditures when seeking care for their injuries - without making care affordable, definitive fracture treatment will never be readily available [8,9].

With local government´s inability to dedicate required resources to improve road safety laws, infrastructure and road safety education, injuries from RTCs will continue to rise as motorized transport becomes the mainstay in the developing world. As in 2015, RTC was the most common mechanism of injury for admitted patients in the current study. Motorcycles, a very common form of inexpensive transport, represented over two-thirds of RTC injuries; patients sustained their injuries either as a driver, passenger or pedestrian. The majority of patients were admitted for fracture care, with nearly 84% having at least one lower extremity fracture and more than 75% were male. Untreated lower extremity fractures confer a great deal of long-term morbidity as the lack of infrastructure result in most LMICs being non-wheelchair accessible. The high prevalence of injured males significantly impacts the Tanzanian economy as most patients do not return to the work force without definitive surgical fixation [9].

Analysis of the retrospective data revealed a 35% increase in the overall orthopaedic disease burden between 2015 and 2018. When assessing the individual components, the ED exhibited a 35.1% decrease in consultations, the outpatient clinic demonstrated a 76.6% increase in the patients evaluated and orthopaedic ward admissions increased by 16.9%. These findings reflect an increase in clinic capacity; KCMC increased the frequency of outpatient clinics from two weekly in 2015 to five weekly in 2018. With the increased outpatient clinic capacity, many patients historically seen in the ED were redirected to the orthopaedic outpatient clinic for evaluation. Over the study period, 1,179 fewer patients were evaluated in the ED in 2018 compared to 2015. Even if these patients were preferentially seen in the outpatient clinic, this transfer did not account for the additional 3,700 patients seen in 2018 compared to 2015. The exhibited increase in inpatient ward admissions represents the overall increased demand for orthopaedic services - the musculoskeletal disease burden at KCMC is persistent and growing. Potential non-epidemiological causes for the increase in musculoskeletal disease burden at KCMC were considered. However, there were no closures of hospitals in the region that provided orthopaedic surgery, nor was there any significant improvement in road infrastructure in the area - both of which might have resulted in an increased burden of disease presenting to KCMC.

Among the strengths of this study is that it provides actual country specific data on the burden of musculoskeletal disease in a LMIC. Such data remains scarce, with most burden of surgical disease estimates extrapolated from indirect methods, such as demographic surveillance systems, household surveys and a combination of models [10,11]. An important limitation to this study is that it only includes patients that present to a tertiary care center - patients in the region that sustain orthopaedic injuries may never seek care due to system constraints (lack of transportation, travel distance and unaffordability). In addition, alternative healers continue to be common in Northern Tanzania. Other sub-Saharan African studies have reported that as many as 85% of people who suffer an acute fracture first visit alternative healers (including traditional “bone setters”) for care [12]. Further work has been undertaken by our research team to more accurately characterize the utilization and role of alternative healers in northern Tanzania. Identifying ways to collaborate with alternative healers will aid in the awareness of allopathic medical centers and the provision of services [13]. Finally, the definition of “definitive treatment” was subjective. If intramedullary nailing or ORIF was performed, the procedure was deemed to be definitive, but the fixation technique, surgical approach, implant used, or other technical criteria were not used to evaluate the reconstruction. Furthermore, definitive treatment does not guarantee a favorable outcome; preliminary investigation into post-operative rehabilitation services available via KCMC have shown this to be an area in need of substantial development. With known resource limitations in implant availability, the percentage of orthopaedic injuries effectively treated with surgery is likely lower than our calculations suggest.

## Conclusion

The burden of orthopaedic surgical disease seen at KCMC is dominated by trauma and is increasing. Significantly fewer available resources leave a growing burden of neglected orthopaedic surgical disease. Without new strategies to address this worsening situation, the discrepancy between supply and demand for musculoskeletal surgical care in the developing world will continue to grow.

### What is known about this topic

- The burden of musculoskeletal disease in low- and middle-income countries (LMIC) continues to increase mainly due to road traffic crashes (RTC);
- In LMICs, even at tertiary referral centers, definitive orthopaedic surgical care is not readily accessible for most patients.

### What this study adds

- An assessment of the current growth rate over a three-year period of musculoskeletal burden of disease at a tertiary referral center in sub-Saharan Africa;
- Insight into the true burden of orthopaedic injuries, taking into account current systems limitations, at a tertiary referral center in sub-Saharan Africa.
